# Proteomic data of the *Trypanosoma cruzi* insect-dwelling epimastigotes overexpressing the RNA-binding protein UBP1

**DOI:** 10.1016/j.dib.2024.110085

**Published:** 2024-01-24

**Authors:** Karina B. Sabalette, Vanina A. Campo, Javier G. De Gaudenzi

**Affiliations:** a Instituto de Investigaciones Biotecnológicas, Universidad Nacional de San Martín - Consejo Nacional de Investigaciones Científicas y Técnicas, 1650 General San Martín, Prov. de Buenos Aires, Argentina; b Escuela de Bio y Nanotecnologías (EByN), Universidad Nacional de San Martín, 1650 General San Martín, Prov. de Buenos Aires, Argentina

**Keywords:** Trypanosomes, Gene expression, Protein profile, Upregulation

## Abstract

We present data on the proteome of the *Trypanosoma cruzi* epimastigote cells overexpressing the U-rich RNA-binding protein 1 (UBP1). The role of this regulatory protein during the epimastigote-to-metacyclic trypomastigote stage transition was clearly established by our group at the transcriptome level; nevertheless, the impact of UBP1 overexpression on protein synthesis is not known. To address this question, we performed shotgun label-free quantification proteomics using an *in vitro* system based on the tetracycline-inducible expression of TcUBP1 and epimastigote wildtype cells. Using tryptic peptide digestion and LC-MS/MS analysis with Orbitrap technology, this data file describes the proteome of three biological samples per condition and yields 1637 correctly quantified proteins. The statistical comparisons of the two analyzed groups within the Proteome Discoverer platform identified 379 differentially expressed proteins, with 207 being up-regulated and 172 being down-regulated. In addition, profile plots and heatmap analysis to visualize the distribution of protein abundances within replicates are also presented. Data are available via ProteomeXchange with identifier PXD047761.

Specifications Table

**Table:** 

Subject	Proteomics. Biochemistry.
Specific subject area	Dataset of the differential proteome of TcUBP1-GFP overexpressing epimastigote cells.
Data format	Raw, Analyzed
Type of data	Table, Figure
Data collection	Protein was extracted from induced and wild-type samples: UBP1-GFP and CTRL cells (three replicates per condition) were analyzed by LC-MS/MS using the Proteome Discoverer platform for statistical and comparative analysis. Raw LC-MS/MS files were made publicly available within the ProteomeXchange Consortium.
Data source location	Buenos Aires, Argentina.
Data accessibility	The mass spectometry proteomics data have been deposited to the ProteomeXchange Consortium via the PRIDE partner repository with the dataset identifier PXD047761 (replicates JDG05, JDG06, and JDG07 for CTRL sample; and replicates JDG08, JDG09, and JDG10 for UBP1-OE sample) (available at https://www.ebi.ac.uk/pride/archive/projects/PXD047761).
Related research article	RNA-seq reveals that overexpression of TcUBP1 switches the gene expression pattern toward that of the infective form of *Trypanosoma cruzi, J BiolChem* (2023) 299(5):104623, https://doi.org/10.1016/j.jbc.2023.104623.

## 1 Value of the Data

• This is the first proteome analysis of *Trypanosoma cruzi* RNA-binding protein UBP1-overexpressing parasites.
• The proteomic dataset underlines the quantitative differences of significant up- or down-regulated proteins as a direct effect of UBP1-overexpression.
• The proteome changes might, in the future, lead to a better understanding of how epimastigote-to-metacyclic form differentiation is orchestrated.

## 2 Background

Extending the dataset previously obtained in the related study by Sabalette et al. [1], here we present proteomic data for insect-dwelling *Trypanosoma cruzi* epimastigotes overexpressing TcUBP1 (TcCLB.507093.220), an RNA-binding protein with post-transcriptional roles in gene expression regulation [2]. We demonstrated that UBP1 induction in the epimastigote form regulates gene expression by affecting mRNA levels. This leads to the up-regulation of genes associated with the trypomastigote infective stage and the downregulation of genes related to ribosomal and synthetic pathway proteins, resembling the transition to the infective metacyclic trypomastigote stage [3]. The current study was initiated due to a lack of understanding regarding the influence of UBP1 expression at the level of protein synthesis.

## 3 Data Description

**Fig. 1:**
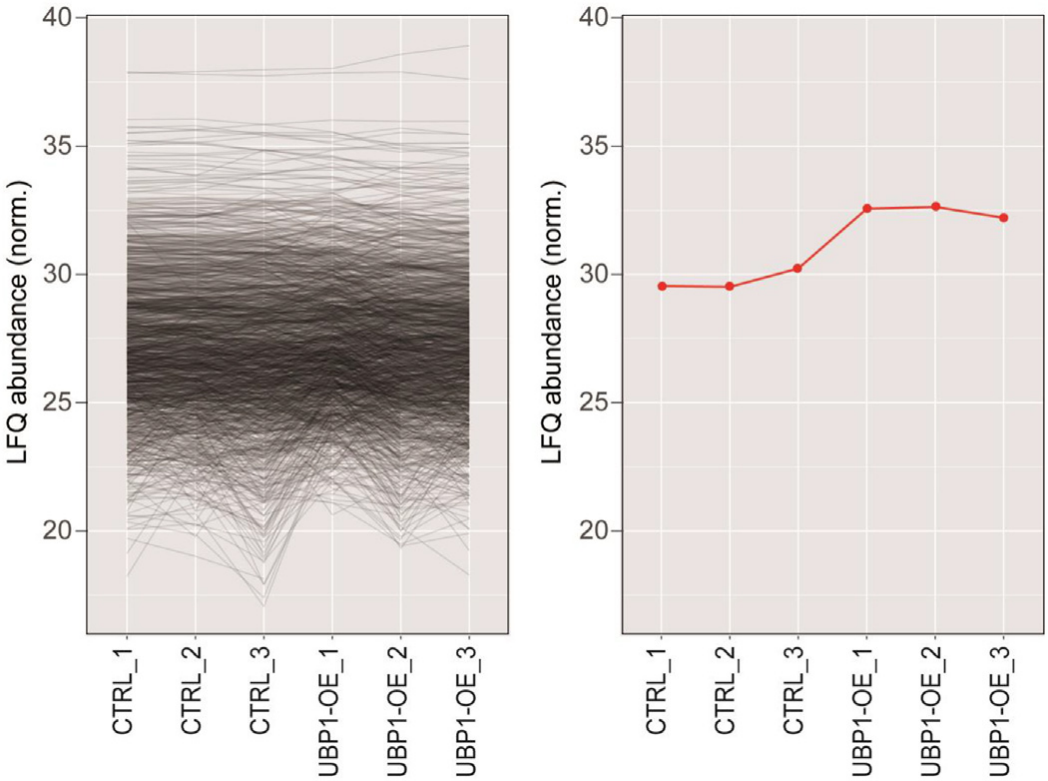
Profile plots showing the trend of LFQ protein abundance identified in the experiment (expressed in Log2 values). *Left*, Representation of the total proteome; *Right*, UBP1-GFP alone. CTRL_1/2/3, control wildtype epimastigotes; UBP1-OE_1/2/3, TcUBP1 induction in epimastigotes.

**Fig. 2:**
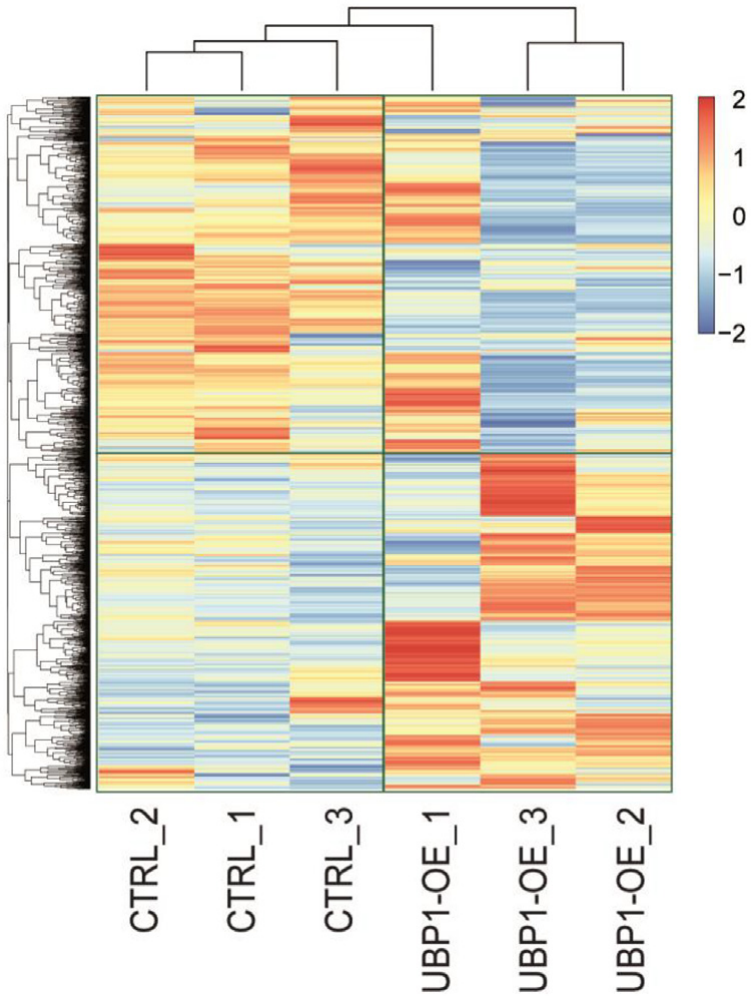
Heatmap of LFQ protein abundances to show the separation of the two analyzed groups (CTRL and UBP1-OE) using pheatmap function in R [4].

**Fig. 3:**
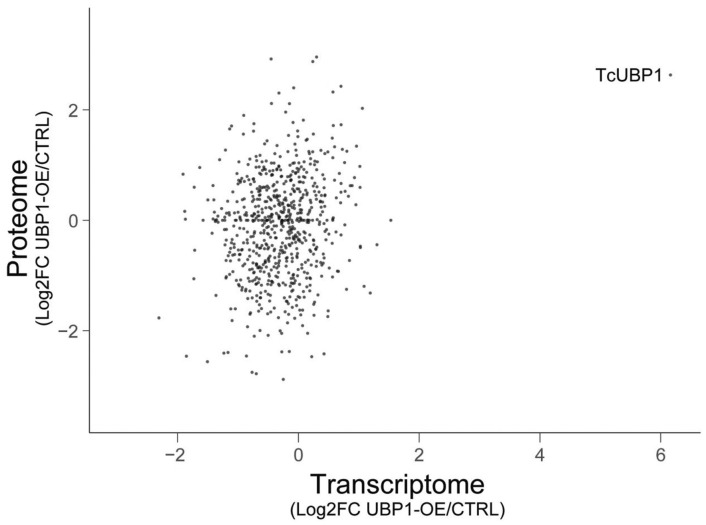
Log-log scatter plot of the expression values in transcriptome (log2 fold change UBP1-OE/CTRL) *vs* proteome experiments (log2 fold change UBP1-OE/CTRL). Expression value for TcUBP1 is marked.

This data consists of a shotgun label-free proteomic experiment to investigate the differential protein expression profiles of *T. cruzi* epimastigote cells overexpressing the TcUBP1-GFP protein [3]. We used two different samples with three biological replicates each, of wild-type RNA-binding protein TcUBP1 and tetracycline-induced epimastigotes for 4 days, CTRL and UBP1-OE, respectively. In more detail, the proteomes of these cells were reduced, alkyated, digested, and analyzed by liquid chromatography-tandem mass spectrometry (LC-MS/MS) using a Q-Exactive mass spectrometer with Orbitrap technology. The obtained spectra were analyzed using the Proteome Discoverer program, utilizing the database specific to the *T. cruzi* (strain CL Brener) UP000002296. The LFQ analysis revealed a total of 1637 correctly quantified proteins over 1749 identified proteins. Of these, 379 proteins were found to be two-fold differentially expressed between UBP1-GFP and CTRL samples: 207 were up-regulated and 172 were down-regulated. Supplementary Table 1 contains a list of the differential proteins characterizing the proteome of UBP1-overexpressing epimastigote cells. The list includes, for each protein: UniProt accession number, systematic gene name, protein description, and fold change value. Then, the datasets were examined to check the amount of UBP1 levels within the two conditions, with UBP1 being the experimental internal control of our proteomic analysis. The profile plots showed in Fig. 1 stress the trend of LFQ abundance of identified proteins and, separately, for UBP1 among samples (CTRL and UBP1-OE replicates). Furthermore, the R software [4] was utilized to create a heatmap that illustrates the distribution of protein abundances among the replicates (Fig. 2). Some differences are observed between replicates, especially for the UBP1-OE_1 sample, and we cannot rule out that this may be due to biological variability within this group. However, in essence, these variations do not stem from different levels of UBP1, as the induction values in the triplicates (Fig. 1, Right) are consistent with those before obtained in our laboratory [3]. While some quantitative proteomics in trypanosomatids have shown that the transcriptome and the proteome positively correlate, it is not always possible to correctly predict protein levels based solely on mRNA levels due to extensive posttranscriptional mechanisms that control gene expression [5,6]. In addition, the proteome correlates much better with the translatome than with the transcriptome [5]. Taking this into consideration, we generated a log-log scatter plot illustrating fold change values for transcriptomic and proteomic experiments using UBP1-overexpressing cells (Fig. 3). We found a positive but low correlation between both datasets (*r* = 0.17). The obtained Pearson correlation coefficient was even lower than *r* = 0.31, which Smircich et al. reported for metacyclic trypomastigote cells [5]. However, it is interesting to note the presence of several downregulated ribosomal proteins in both approaches (in particular, 35 out of the 172 downregulated proteins in Supplementary Table 1 are related to the translation process). This result is consistent with previous findings from RNA-Seq experiments [1] and with parasites exhibiting an expression profile similar to the infective form. Finally, it is noteworthy that along with UBP1, other RNA-binding proteins are also up-regulated (TRRM1A, TcCLB.509317.60 [7]; ZC3H39, TcCLB.508895.50 [8]; and snoRBP, TcCLB.507649.80 [9]), so we cannot ignore that the observed effect in UBP1-overexpressing epimastigotes may result from a regulatory cascade involving these proteins as well.

## 4 Experimental Design, Materials and Methods

### 4.1 Cell sample preparation and LC-MS/MS analysis

TcUBP1 was inducibly expressed in the *T. cruzi* CL Brener epimastigote strain as described [3]. Control wild-type UBP1 and four-day tetracycline-induced parasites were cultured in epimastigote form at 28 °C. Proteins were extracted and analyzed by mass spectrometry at the Protemics Core Facility of the CEQUIBIEM, Facultad de Ciencias Exactas y Naturales, Universidad de Buenos Aires, Argentina. The protein bands that were excised from the Coomassie blue-stained SDS-PAGE gels were washed and destained using a series of solutions: 50 mM ammonium bicarbonate, 25 mM ammonium bicarbonate, 50% acetonitrile, and 100% acetonitrile. Then, protein bands were reduced with 20 mM dithiothreitol for 45 min to 56 °C and alkylated with 50 mM iodoacetamide for 45 min. After that, the proteins were in-gel digested with 100 ng trypsin (Promega V5111) in 25 mM ammonium bicarbonate overnight at 37 °C. The peptides were recovered by elution with 50% acetonitrile and 0.5% trifluoroacetic acid. This extraction process included brief sonication, and then the solution was further concentrated by using a speed-vacuum drying method. The samples were diluted in 15 µL of water with 0.1% formic acid, purified using C18 ZipTip (Merck Millipore), and extracted with 10 µL of a mixture of water, acetonitrile, and formic acid in a ratio of 40:60:0.1%. The digests were analyzed using nanoLC-MS/MS on a Thermo Scientific Q-Exactive Mass Spectrometer, which was coupled with a nanoHPLC EASY-nLC 1000 (Thermo Scientific). For LC-MS/MS analysis, approximately 2 micrograms of peptides were introduced into a reverse-phase column with the following specifications: C18, 2 micrometers, 100 Å, 50 micrometers in diameter, and 150 mm in length. The Easy-Spray Column PepMap RSLC (P/N ES801) is designed for effectively separating protein complexes with exceptional resolution. The nano-column was operated at a flow rate of 300 nanoliters per minute, with the solvent composition ranging from 7% B for 5 min to 35% B for 120 min. Solvent A consisted of water with a 0.1% concentration of formic acid, while solvent B consisted of acetonitrile with a 0.1% concentration of formic acid. The volume of the injection was 2 microliters (µL). An electric potential of 1.5‒3.5 kV was applied for the process of Electrospray Ionization using the Thermo Scientific EASY-SPRAY system. Full-scan mass spectra were obtained using an Orbitrap analyzer, covering a mass range of 400‒2000 *m/z* at a resolution of 70,000 at 400 *m/z*. In each cycle, the twelve most intense ions were consecutively isolated, subjected to higher-energy collision dissociation for fragmentation, and then measured in the Orbitrap analyzer. Peptides with a charge of + 1 or with an undetermined charge state were excluded from MS/MS fragmentation.

### 4.2 Proteomic analysis

The raw data from the Q-Exactive mass spectrometer was subjected to analysis using the Proteome Discoverer software (version 2.2, Thermo Scientific) and searched against the freely available database of the *T. cruzi* CL Brener strain (Organism ID: 353153, Proteome ID: UP000002296, total proteins: 19,245) with trypsin specificity, allowing a maximum of two missed cleavages per peptide. Carbamidomethylation of cysteine residues was assigned as a fixed modification, and methionine oxidation was considered a variable modification. Searches in Proteome Discoverer utilized a precursor mass tolerance of 10 ppm and a product ion tolerance of 0.05 Da. Protein hits were filtered based on high-confidence peptide matches with a peptide false discovery rate of 1%, calculated using a reverse database strategy.

## Limitations

Not applicable.

## Ethics Statement

The authors have read and follow the ethical requirements for publication in Data in Brief and confirming that the current work does not involve human subjects, animal experiments, or any data collected from social media platforms.

## Declaration of Generative AI and AI-Assisted Technologies in the Writing Process

During the preparation of this work the authors used ChatGPT (OpenAI) in order to improve language and readability. After using the tool/service, the authors reviewed and edited the content as needed and take full responsibility for the content of the publication.

## CRediT authorship contribution statement

**Karina B. Sabalette:** Methodology. **Vanina A. Campo:** Methodology, Formal analysis. **Javier G. De Gaudenzi:** Formal analysis, Visualization, Writing – original draft, Conceptualization, Funding acquisition, Supervision.

## Data Availability

Proteomic data of the Trypanosoma cruzi RNA-binding protein UBP1 induction and knockdown in insect-dwelling epimastigotes (Original data) (PRIDE).

## Appendix Supplementary materials

Table S1 List of 379 affected proteins after UBP1-GFP overexpression with ≥2-fold change. Of these, 207 proteins were up-regulated and 172 were down-regulated. Image, application 1
